# Nonequilibrium Phonon Dynamics and Its Impact on the Thermal Conductivity of the Benchmark Thermoelectric Material SnSe

**DOI:** 10.1021/acsnano.3c03827

**Published:** 2023-10-20

**Authors:** Amit Kumar Prasad, Jakub Šebesta, Raquel Esteban-Puyuelo, Pablo Maldonado, Shaozheng Ji, Biplab Sanyal, Oscar Grånäs, Jonas Weissenrieder

**Affiliations:** † Materials and Nano Physics, School of Engineering Sciences, KTH Royal Institute of Technology, SE-100 44 Stockholm, Sweden; ‡ Materials Theory, Department of Physics and Astronomy, Uppsala University, Box 516, 751 20 Uppsala, Sweden

**Keywords:** Photoinduced electron diffuse scattering (PDS), thermoelectric, nonequilibrium phonon dynamics, SnSe, Ultrafast electron microscope (UEM), electron−phonon coupling, phonon−phonon scattering

## Abstract

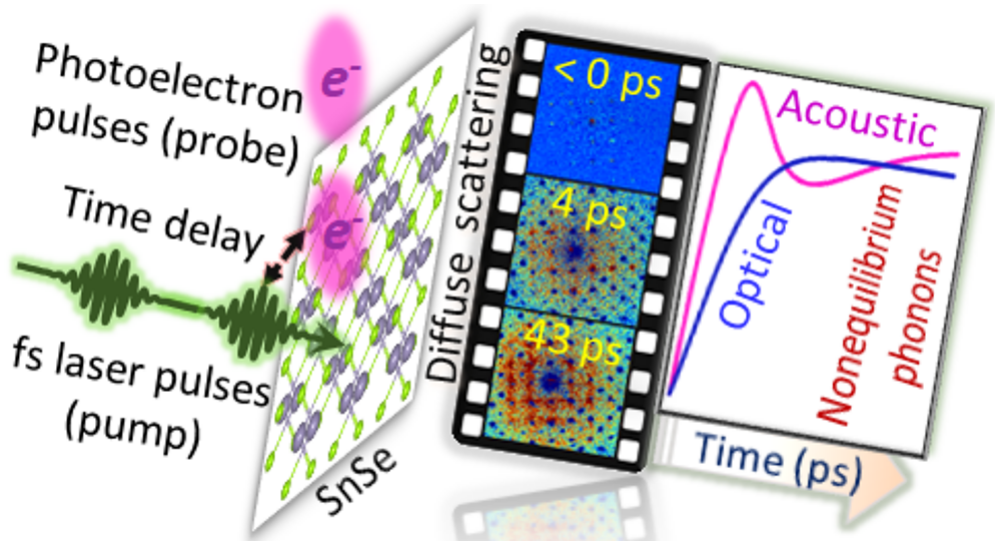

Thermoelectric materials play a vital role in the pursuit of a sustainable energy system by allowing the conversion of waste heat to electric energy. Low thermal conductivity is essential to achieving high-efficiency conversion. The conductivity depends on an interplay between the phononic and electronic properties of the nonequilibrium state. Therefore, obtaining a comprehensive understanding of nonequilibrium dynamics of the electronic and phononic subsystems as well as their interactions is key for unlocking the microscopic mechanisms that ultimately govern thermal conductivity. A benchmark material that exhibits ultralow thermal conductivity is SnSe. We study the nonequilibrium phonon dynamics induced by an excited electron population using a framework combining ultrafast electron diffuse scattering and nonequilibrium kinetic theory. This in-depth approach provides a fundamental understanding of energy transfer in the spatiotemporal domain. Our analysis explains the dynamics leading to the observed low thermal conductivity, which we attribute to a mode-dependent tendency to nonconservative phonon scattering. The results offer a penetrating perspective on energy transport in condensed matter with far-reaching implications for rational design of advanced materials with tailored thermal properties.

## Introduction

Through conversion of waste heat gradients into electric current,^1−5^ thermoelectric devices may serve an important role in realizing a sustainable energy system.^1−9^ The efficiency of a thermoelectric material is governed by its figure of merit *zT* = *S*^2^σ*T*/*k*, where σ is the electrical conductivity, *k* is the thermal conductivity, which is the sum of the electrical (*k*_e_) and lattice (*k*_l_) contributions, *T* is the absolute temperature, and *S* is the Seebeck coefficient.^1−5^ Therefore, for a material to exhibit high *zT*, it must possess low *k* in combination with high σ and *S*. However, optimizing thermoelectric properties is challenging since the relevant material parameters are interconnected through carrier concentration, mobility, and phonon characteristics. Several strategies have been suggested in the literature to improve *zT*.^3−8^ These strategies can be classified into two categories: (a) band engineering, which is associated with increasing the electrical transport, and (b) phonon engineering, which is associated with enhancing phonon scattering to control the thermal transport.^5−8^ Recently, single-crystalline tin selenide (SnSe) was reported to exhibit a *zT* ∼ 2.6 along its crystallographic *b*-axis at 923 K (see Figure S1 for crystal structure).^9^ The high *zT* was attributed to an ultralow thermal conductivity of the lattice ∼ 0.25 W/mK.^9^ The combination of excellent *zT*, low cost, and nontoxic composition from highly abundant elements makes SnSe a promising material system for future applications. Efforts have been focused on further enhancing the *zT* and in understanding the mechanism behind the anisotropic ultralow thermal conductivity in the *a*, *b*, and *c* directions of SnSe.^10−14^ Recent studies have indicated that an intrinsic strong anharmonicity in the Sn–Se bond, which enhances anharmonic phonon scattering processes, is central to the ultralow thermal conductivity in SnSe.^9,10,12−14^ However, the fundamental mechanisms underpinning such scattering processes and how they govern the microscopic thermal energy transport in SnSe remain unclear.

Photoexcitation using ultrashort laser pulses has been successfully deployed to transiently control the dynamics of electron and/or phonon populations.^15−17^ This approach has significant potential in unraveling the operating mechanisms of thermoelectric devices, as they operate under temperature gradients, i.e., at nonequilibrium states. However, understanding out-of-equilibrium properties is challenging for both theory and experiments due to the demanding nature of modeling nonequilibrium states and the intrinsic spatiotemporal scales at which relevant processes occur. While the phonon subsystem dominates thermal transport in SnSe,^12−14,18,19^ it remains sensitive to phonon interactions with the electronic system. Therefore, it is essential to gain a comprehensive understanding of the nonequilibrium dynamics of electron–phonon (e-ph) and phonon–phonon (ph-ph) interactions to develop predictive capabilities for fine-tuning the thermoelectric response in materials and enable future technological applications.^20,21^

Ultrafast pump–probe spectroscopy^13^ and Raman spectroscopy^10^ are experimental techniques that offer valuable, albeit indirect, insights into nonequilibrium anharmonic phonon scattering and thermally excited phonon modes at the gamma (Γ) point in the Brillouin zone (BZ). Inelastic neutron scattering, on the other hand, can provide information on the phonon scattering process throughout the entire BZ.^14^ However, these techniques have limitations in terms of either time or momentum resolutions, which prevent complete analysis of the nonequilibrium mode-resolved phonon dynamics across the momentum space. A combination of time and momentum resolutions is necessary to provide a comprehensive understanding of nonequilibrium phonon dynamics over the entire momentum space, which is essential for developing predictive models of thermoelectric materials for future technological applications.

Ultrafast electron diffraction (UED) can provide valuable insights into ultrafast lattice dynamics in nonequilibrium states following ultrashort photoexcitation.^21−33^ However, to fully comprehend the dynamics of e-ph and ph-ph interactions, it is necessary to analyze the entire momentum space, which includes both the Bragg spots and the diffuse scattering (DS) present between them.

The importance of this approach has been highlighted by recent studies, which have shown its ability to facilitate the investigation of fundamental excitations and their dynamic interactions in both temporal and momentum spaces.^18,19,25−33^ In particular, related to the present work, this advanced technique allows for valuable insights into the nature of e-ph coupling in relation to the bimodal polaron formation in SnSe.^18^

Furthermore, the relation between the experimentally measured photoinduced diffuse scattering (PDS)^25−33^ to the transient momentum-dependent phonon population, and ultimately thermal conductivity, can be resolved through nonequilibrium kinetic theory.^33−35^ Previous work has shown that rate equations parametrized for first-principles calculations,^33,34^ coupled to the explicit calculation of the PDS patterns as a function of time, provide a powerful tool for elucidating relaxation pathways of excited carriers.^33^ By calculating the PDS signal for each phonon mode,^36^ we may determine the time-dependent PDS based on individual phonon populations acquired from nonequilibrium kinetic theory. Leveraging computational techniques, we performed an in-depth analysis of the ph-ph scattering mechanisms, providing us with a direct avenue to link the measured PDS to the thermal conductivity of SnSe.

In this study, we utilize the PDS^25−33^ and temperature-induced diffuse scattering (TDS)^36,37^ capabilities of an ultrafast transmission electron microscope (UEM),^38^ along with nonequilibrium kinetic theory analysis,^33−35^ to investigate the momentum-dependent phonon dynamics in SnSe. The combination of experiment and theory renders momentum-dependent phonon dynamics in SnSe accessible. This is relevant since the experiment alone is sensitive only to the total DS intensity. The results provide a detailed description of mode-resolved phonon dynamics and energy redistribution processes. This is crucial for the understanding of thermal energy transport immediately after an excitation event such as an increase in temperature. The obtained nonequilibrium phonon populations suggest that the phonon modes with significant nonconservative phonon scattering are highly populated and will be substantial contributors to the low thermal conductivity in SnSe. These findings uncover the interplay between the electronic and phononic subsystems and offer a fundamental understanding of the thermoelectric properties of SnSe.

## Results and Discussion

### Temperature Dependent Diffuse Scattering

**Figure 1:**
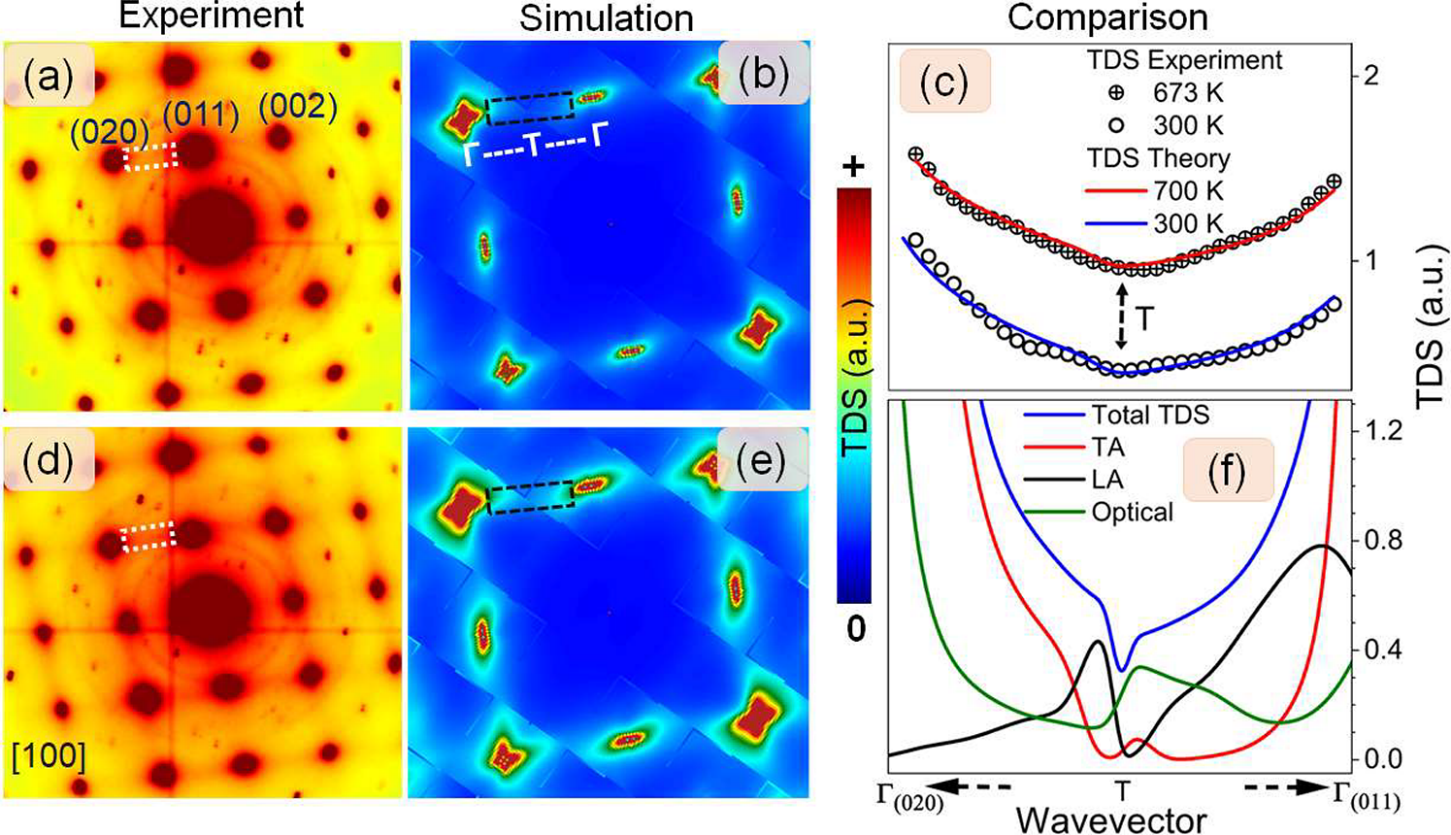
Comparison between experimental and simulated temperature-induced diffuse scattering (TDS) of SnSe at the [100] zone axis. Experimental electron diffraction patterns collected at (a) 300 and (d) 673 K. Simulated total TDS at (b) 300 and (e) 700 K. (c) Experimental and calculated TDS line profiles between Γ_(020)_ and Γ_(011)_ (regions extracted for line of profiles are indicated by white dotted rectangles in the experimental results and black dashed rectangles in the simulated results)_._ The black dashed arrow indicates the BZ boundary (T). (f) Phonon branch contributions to the total TDS at 300 K between Γ_(020)_ and Γ_(011)_ highlighting contribution at T. The arrows at the abscissa pointing toward Γ_(020)_ and Γ_(011)_ indicate that the TDS intensity in the vicinity of Bragg spots was excluded in (c) and (f).

Projections of the crystal structure of the relevant *Pnma* phase of SnSe, along selected crystallographic directions, are shown in Figure S1 (Supporting Information).^39^Figure 1a,d shows [100] zone axis electron diffraction patterns collected at 300 and 673 K. The patterns are presented on a false-color scale saturating the Bragg peaks to enhance the TDS contrast. The saturation of the Bragg peaks allows for the detection of additional symmetry forbidden weak spots from rare events of multiple electron scattering. For reference, a diffraction pattern with Bragg peak optimized contrast is shown in Figure S4b. The sharpness of the Bragg peaks demonstrates the single-crystalline nature of the sample. The overall background intensity (TDS) increases with the sample temperature. Interestingly, the TDS exhibits distinct signatures observable as streaks connecting neighboring low-order Bragg spots, e.g., (020) and (002) (e.g., between the lattice planes {020} and {002}). The intensity of the streak patterns increases with the temperature, but the shape does not change over the analyzed temperature range.

The TDS results provide momentum resolved information on superimposed contributions from thermally excited phonon modes.^36,37^ However, interpretation of the experimental results in the context of mode dependent contribution is strenuous. In order to determine which phonon modes are responsible for the TDS signature, we performed ab initio calculations of the SnSe phonon band structure (Figure S2A) and simulated branch-dependent TDS contributions related to thermally excited phonon populations (Figure S3). The TDS was calculated in the first order at similar temperatures to the experimental conditions.^36^ In the simulated TDS patterns, presented in Figure 1b,e, we observe a streak pattern signature similar to that in the experimental results. Figure 1c shows a comparison of the experimental and simulated line profiles extracted between Γ_(020)_ and Γ_(011)_ at the two selected temperatures. Here, it is worth mentioning that as we approach the BZ center (Γ point), the experimental results are dominated by the intensity of the Bragg spots, and therefore, the regions around Γ are excluded from the line profiles.

Decomposition of the total TDS into phonon mode contributions shows that the acoustic modes are responsible for the observed line profile asymmetry (Figure 1f), particularly the transversal acoustic (TA) mode TA_2_ (Figure S3), the polarization vector of which lies in the studied k-plane. TA_2_ dominates the total TDS intensity near the BZ center (Γ); however, near the BZ boundary (T, at the midpoint between Γ_(020)_ and Γ_(011)_) its intensity decreases asymmetrically, approaching negligible contribution. Contributions from other modes, such as longitudinal acoustic (LA) or optical modes (OPT), are less significant to the observed asymmetry. Note, in our experimental geometry, we are not sensitive to the TA_1_ mode as it predominantly points out of the studied plane (see structure factor, eq 5 in Methods section). The modes are assigned using a naming convention based on the character near the Γ_(000)_ point (Figure S2A). The character of the phonon modes can change along the Γ-T path; e.g., the TA_2_ mode experiences a loss of transversal character in proximity to the T-point. Structure factor consideration (eq 5, Methods section) implies that the change in character will lead to suppression of the intensity along the studied path. Note, the polarization vectors in the one-phonon structure factor (eq 5) will be modified by a phase factor outside the first BZ.

When analyzing the line profile of simulated TDS along the Γ_(020)_–T−Γ_(011)_ path, certain interesting observations emerge (Figure 1b,e). The tails of the TDS extend from the Γ_(020)_ and Γ_(011)_ points toward the T-point. These tails are not pointing directly at each other (i.e., collinear), but instead, they exhibit a slight tilt. This peculiar behavior can be attributed to the influence of transversal modes in the system. Geometrical considerations imply that the intensity of the TDS along the chosen path is highly sensitive to these transversal modes. Particularly, when we approach the T-point, an important factor comes into play: the dot product in the one-phonon structure factor (eq 5). This dot product results in a maximum value centered outside the line connecting the Γ_(020)_ and Γ_(011)_ spots. The significance of this finding lies in explaining the observed asymmetry in the line profiles (Figure 1c–f).

### Ultrafast Photoinduced Diffuse Scattering

We now focus on ultrafast lattice dynamics induced through excitation by a femtosecond laser pulse. Laser excitation drove the sample into a transient (nonequilibrium) state. The nonequilibrium structural dynamics of the sample can be analyzed by tracing the temporal evolution of the Bragg spot intensities.^25−33^Figure S4c shows the temporal response of the Bragg diffraction intensity at an incident fluence of 2.4 mJ/cm^2^ (experimental details can be found in the Methods section). The temporal evolution of the Bragg spots intensities was evaluated by integrating a circular region (∼4 × 10^–4^ Å^–2^) centered around the Bragg spot of interest and normalized to the intensity before time zero. After photoexcitation, we observe a decrease of approximately 3–15% in Bragg peak intensities at time delays of less than 10 ps. The temporal evolution of the (0*k*0) and (00*l*) Bragg reflections was analyzed using a single-exponential function, returning similar decay time constants of 2.63 ± 0.38 and ∼2.55 ± 0.41 ps. It is worth noting that these time constants are comparable to those reported in a previous study using 800 nm photoexcitation.^19^ However, in contrast to the findings in ref (18) we could not observe sub-picosecond dynamics. Since we use longer probe pulses (∼1.2 ps), this is expected, but excitation using 515 nm may also lead to different relaxation pathways.

The decrease in Bragg intensities with the reciprocal lattice vector **q** at time delays >10 ps (Figure S4d) is consistent with the Debye–Waller (DW) model^23,24^ or suggests that the Bragg peaks at these time delays exhibits an intensity distribution similar to what is expected from an increase in lattice temperature.^23,24^

**Figure 2:**
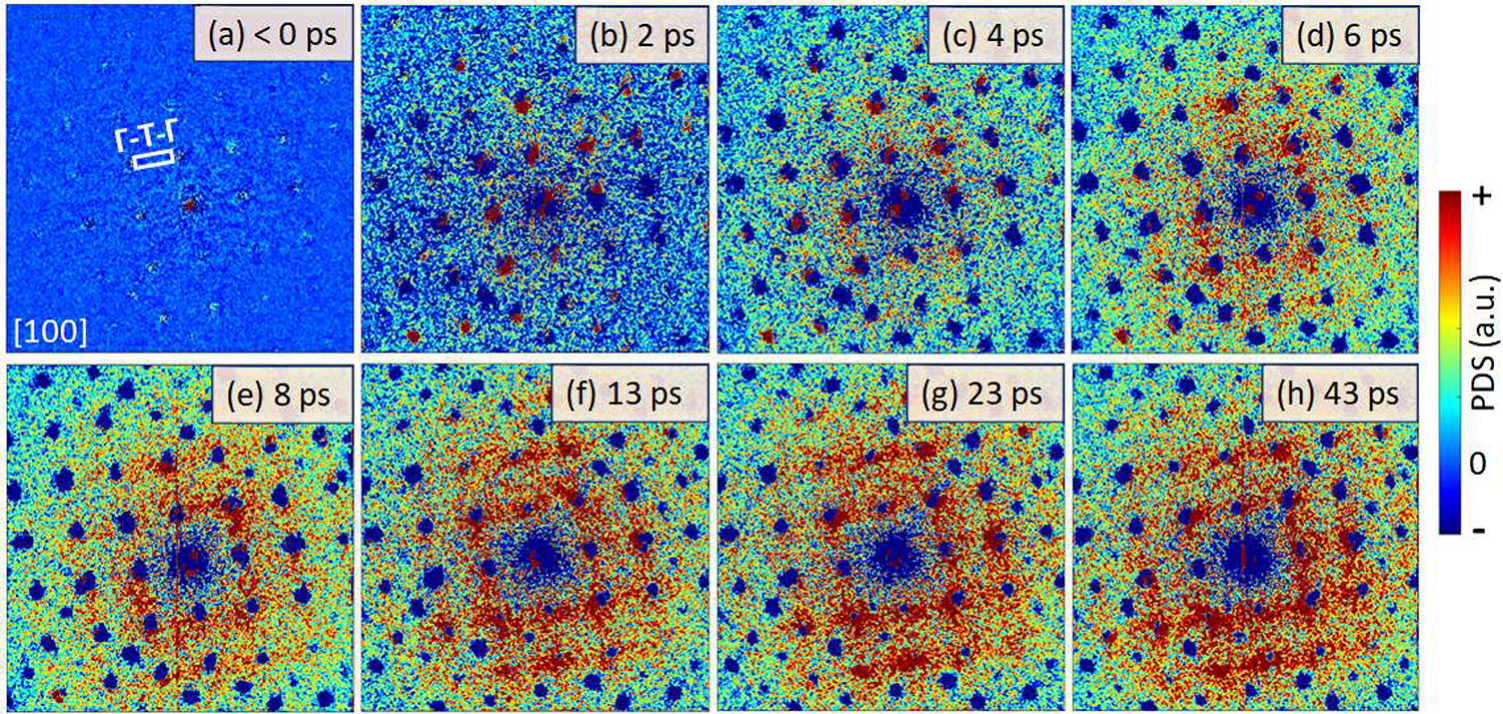
Temporal evolution of photoinduced diffuse scattering (PDS). The PDS results are obtained by subtracting an averaged electron diffraction pattern of the unpumped sample (before time zero) from the diffraction patterns collected at the specified pump–probe time delays at a photoexcitation fluence of 2.4 mJ/cm^2^, as shown in the figures: (a) <0 ps, (b) 2 ps, (c) 4 ps, (d) 6 ps, (e) 8 ps, (f) 13 ps, (g) 23 ps, and (h) 43 ps. Negative intensity (dark blue) appears at Bragg spot positions for positive time delays in accordance with what is expected from the DW model.

The Bragg peaks, however, do not include all available information on the lattice dynamics. Analysis of the PDS between the Bragg spots allows for obtaining momentum-resolved information related to the phonon dynamics.^18,26−33^Figure 2a–h presents PDS results at time delays of <0, 2, 4, 6, 8, 13, 23, and 43 ps after a 515 nm (2.4 eV) laser excitation at a pulse fluence of 2.4 mJ/cm^2^. The relative intensity of the PDS is approximately 4 orders of magnitude weaker compared to that of the Bragg spots. To visualize the PDS structure, we subtracted an averaged UED diffraction pattern at a negative time delay from all UED patterns. At positive delay, the Bragg spots decrease in intensity (DW behavior, Figure S4c,d) and appear as negative (blue) intensities. Due to the low count rates in the PDS analysis, extensive acquisition times were necessary to obtain adequate signal-to-noise ratios (∼several hours of acquisition at each time delay, given our ∼3 detected electrons per pulse on the detector).

Following the laser excitation, we observed an increase of isotropic DS intensity (Figure 2b–d) followed by formation of two rectangular PDS streak patterns with time-dependent intensity (Figure 2d–h). The PDS streaks connect low-index Bragg spots (020) and (002) or (040) and (004) (e.g., between lattice planes {020} and {002} or between lattice planes {040} and {004}). Note that the PDS streak signature resembles the previously observed signature in the TDS patterns (Figure 1a–d).

### Mode-Resolved Nonequilibrium Phonon Dynamics and Energy Transfer

**Figure 3:**
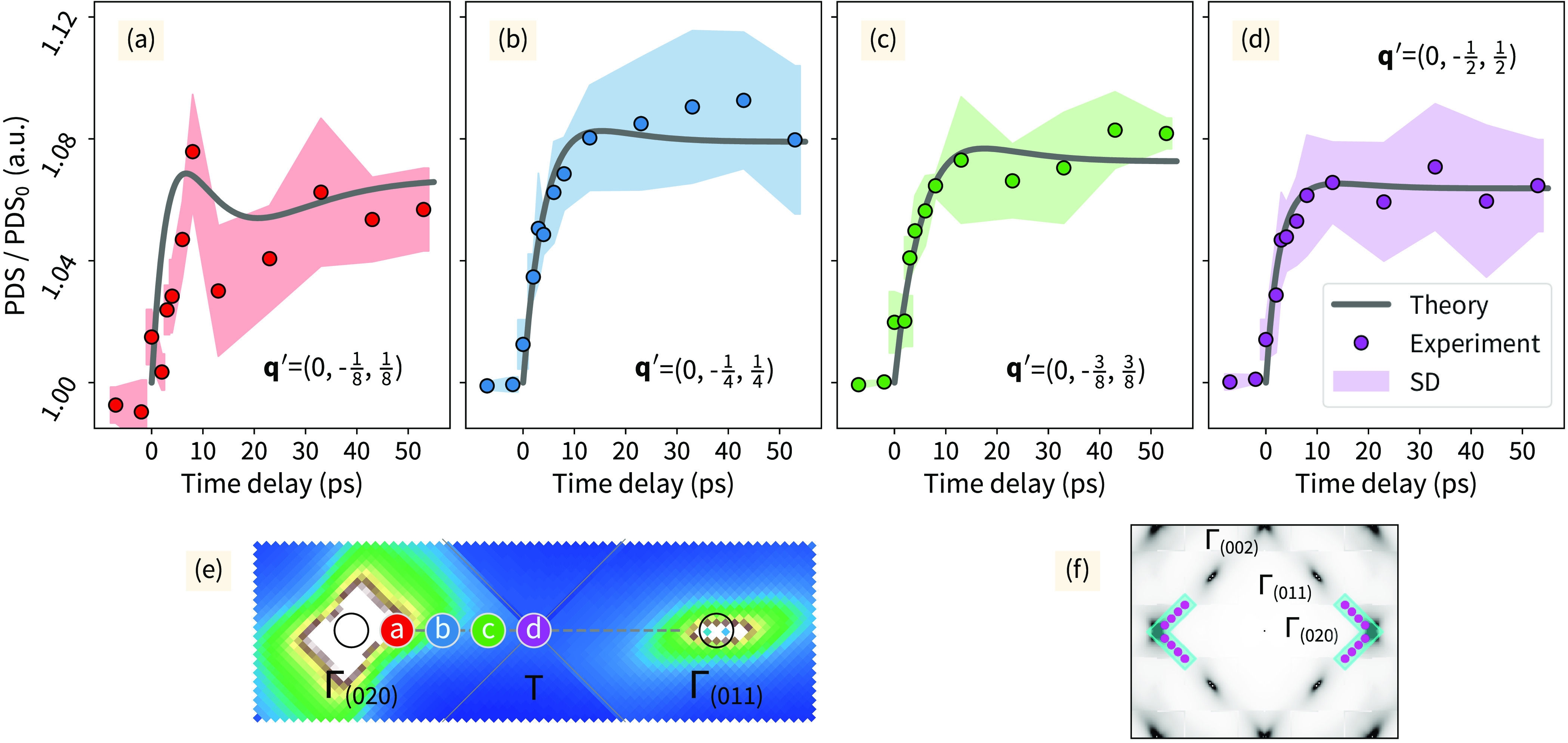
Momentum-resolved temporal dependence of experimental photoinduced diffuse scattering (PDS) in comparison with nonequilibrium kinetic theory simulations. Relative change in PDS with normalized to the PDS_0_ at time delays <0 ps results were analyzed at four different momentum-space positions: (a) **q**′ = (0, −1/8, 1/8), (b) **q′** = (0, −1/4, 1/4), (c) **q′** = (0, −3/8, 3/8), and (d) **q′** = (0, −1/2, 1/2) along the Γ_(020)_ – T – Γ_(011)_ path. Here, experimental PDS (data points), standard deviations (SD, colored regions) of the experimental data points, and simulated PDS (solid lines) are represented. (e) Positions of the selected **q′** points projected on the simulated PDS pattern. (f) Symmetric k-space areas used for averaging the experimental data.

To explore the dynamics in more detail, we inspected PDS snapshots at four distinct **q′** points along the Γ_(020)_ – T – Γ_(011)_ path (Figure 2). The **q′** points are represented as **q′** = **q** – **G**, where **q′** is the relative position with respect to nearest Bragg spot (**G**), and **q** is the scattering vector. We selected **q′** with respect to Γ_(020)_ as follows: {(0, −1/8, 1/8) or 12.5%; (0, −1/4, 1/4) or 25%; (0, −3/8, 3/8) or 37.5%; and (0, −1/2, 1/2) or 50% (T) of the reciprocal distance along Γ_(020)_ – T – Γ_(011)_}. The experimental results (solid circles in Figure 3) were integrated, at each selected **q′** point, over an area of 2 × 10^–4^ Å^–2^ and averaged over symmetrical **q′** points (as depicted in Figure 3f). We conducted a similar analysis as presented in Figure 3 for all **q′** points along the Γ _(040)_ – T – Γ _(031)_ path, which can be found in Figure S5. The PDS dynamics close to the Bragg spots can be influenced by the Debye–Waller response. In our experiments, this contribution becomes significant at **q′** values around (0, −1/10, 1/10). Therefore, we analyzed the PDS data adjacent to the Bragg spots (020) and (040), focusing on specific **q′** values: (0, −1/8, 1/8), (0, −1/7, 1/7), and (0, −1/6, 1/6). At each of these selected **q′** points, we integrated the experimental data over an area of 0.67 × 10^–4^ Å^–2^ and subsequently averaged them across symmetrical **q′** points, as illustrated in Figure S6. The **q′**-dependent PDS data indicate the presence of two distinct dynamical processes. A fast monotonic increase in PDS intensity before time delays of 10 ps was followed by a rather complex, **q′**-dependent behavior over the remaining analyzed pump–probe time delays. Despite the proximity of **q′** = (0, −1/8, 1/8) to the Bragg spot, we were able to observe a distinct local minimum at an approximately 13 ps time delay. The minimum persists until **q′** = (0, −1/6, 1/6). At greater time delays, the intensity monotonically increases (see Figure 3a, Figure S5a, Figure S6a,b).

No analogous dynamics were observed for the other three selected **q′**-points, **q′** = (0, −1/4, 1/4), **q′** = (0, −3/8, 3/8), and **q′** = (0, −1/2, 1/2) (Figure 3b–d and Figure S5b–d). For these **q′**-points, the PDS intensity increases until 10 ps, but no minima could be detected above the uncertainty at later time delays. Instead, the PDS intensity remains approximately constant (Figure 3b–d).

The experimental PDS intensity represents a superposition of all contributing phonon amplitudes according to the one-phonon structure factor (eq 5). This makes deconvolution of the PDS results into mode-resolved dynamics challenging. In order to untangle the dynamics, we compared the experimental PDS results at the selected **q′** points (Figures 3 and S5–S7) with PDS simulations based on nonequilibrium kinetic theory (method section) of ultrafast electron and phonon dynamics.^33−35^ In the simulation, the electrons and phonons are represented as two subsystems interacting via e-ph and ph-ph couplings, controlling the nonequilibrium dynamics.

The e-ph coupling parameters calculated purely from first principles provided a too-rapid system response at early time delay (<10 ps), manifested in an earlier onset of the PDS than the experimentally observed (∼10 ps) at most of the studied **q′** points (Figure S7 – model T1). Therefore, an artificial scaling factor was applied to follow the experimental behavior. Note, this is the only fit parameter in the employed model (see Figure 3, Figure S5, and Figure S6).

**Figure 4:**
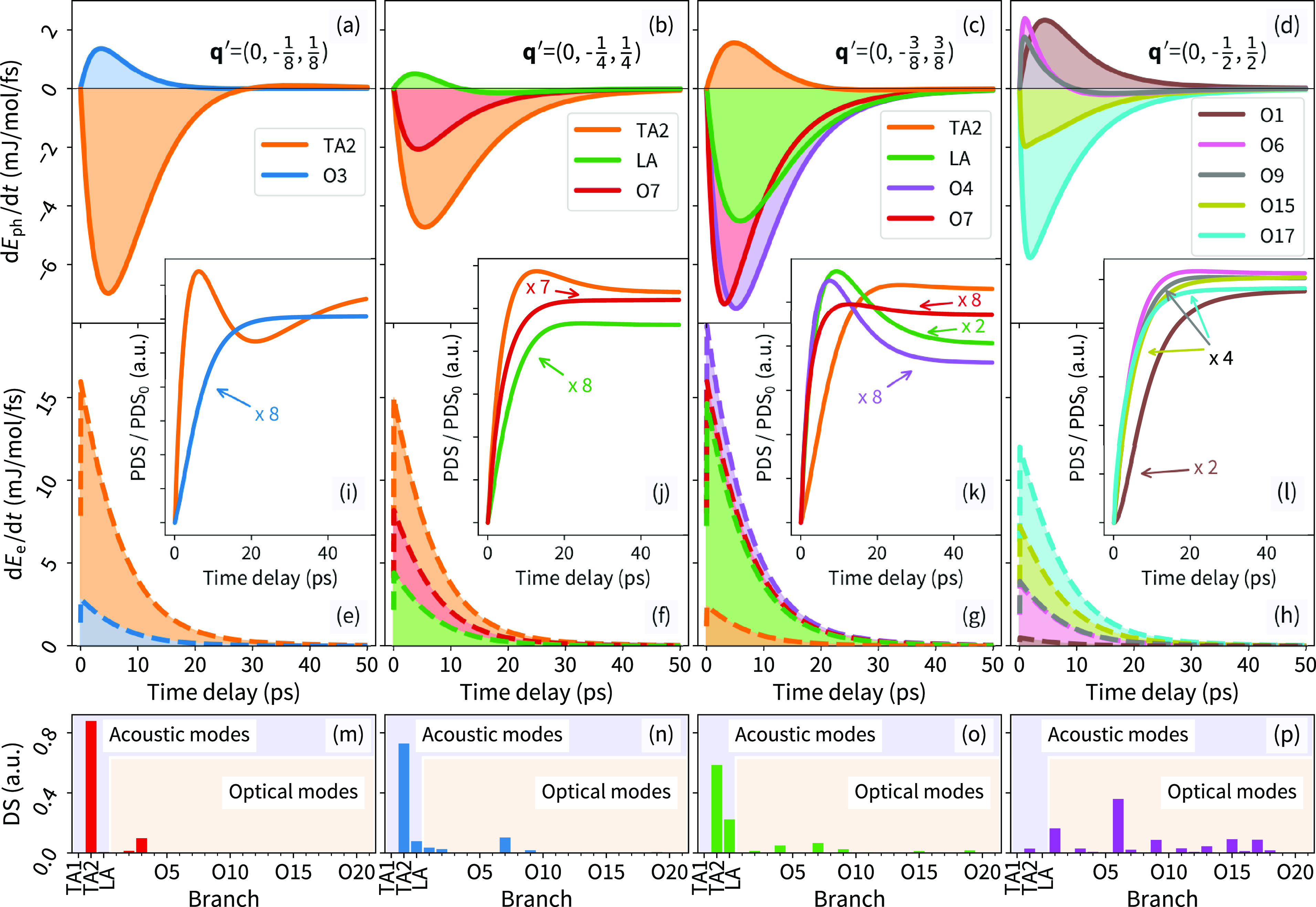
Momentum and phonon mode-dependent mapping of nonequilibrium energy redistribution rates (a–d) colored solid lines from phonon subsystems,  and (e–h) colored dashed lines from electron subsystems, . (i–l) Evolution of the related mode resolved change in PDS intensity with the respect to PDS intensity at time delay <0. Only the most contributing phonon modes were selected at particular **q′** points. (m–p) Simulated mode-dependent relative thermal diffuse scattering (TDS) intensity. The results are related to the four distinct **q′** points presented in (a) to (d). The character of the phonon modes is resolved only for the acoustic modes (TA_1_, TA_2_, LA), and the optical modes are numbered in ascending frequency at Γ_(000)_ (O1–O21). Positive values of nonequilibrium energy redistribution rates are related to an increased population of a phonon mode. For a comparison of the energy flow magnitudes, refer to Figure S9.

Additionally, the exploration of momentum and phonon mode-dependent nonequilibrium energy redistribution rates suggest that e-ph energy flows are dominant at early times (<7 ps) (Figure 4e–h) and are responsible for most of the PDS evolution during this temporal range. The adjustment of the e-ph parameters allowed us to perfectly mimic the initial onset of the PDS intensity (Figures 3, S5, and S6). However, at larger time delays, ph−ph scattering becomes increasingly important. The simulated PDS intensity was rescaled to fit the experimental magnitudes. This is motivated by the normalization of the experimental PDS results to the PDS intensity prior to time zero. Figure S7 displays representative sets of theoretical PDS curves, including the simulations based on the originally calculated e-ph parameters (T_1_ model) as well as on the final fit (Figure 3, Figures S5 and S6), which was selected for further analysis (T_4_ model).

A comparison of the experimental results with the nonequilibrium kinetic model provides an avenue to obtain insights into the mechanism for microscopic energy transfers (see eqs 1 and 2). The model allows us to extract energy transfer rates between the electrons and phonons  (Figure 4e–h) as well as between the phonon modes themselves  (Figures 4a–d) and show the time-dependent energy redistribution within the phononic subsystem. This provides an opportunity to understand the spatiotemporal evolution in PDS. Due to the large size of the phononic system (24 phonon modes), only phonon branches with significant contribution to the total PDS at a particular **q**′ point are selected (Figure 4i–l,m–p). We note that only the acoustic branches can be resolved into a transversal or longitudinal character. As a result of the rather low crystal symmetry, the character of some optical branches is not well-defined (Figure S8). Particularly, the character changes along the [010] and [001] directions near the Γ point. Therefore, we simply number the optical modes in ascending frequency at the Γ point (Figure 4).

As previously mentioned, the nonequilibrium kinetic model analysis shows that the energy flow from the electronic system to the phonon system dominates over the ph-ph scattering at short time delays (<7 ps). This is also reflected in the increase of PDS intensity (up to ∼10 ps) as phonon modes are becoming more populated (Figure 3, Figure S5, and Figure S6). However, the e-ph energy flow decays with time (<10 ps), and the phonon population begins to be significantly affected by phonon scattering processes redistributing delivered energy among other phonon modes at different **q′** points (Figure 4). This is manifested by a flattening of the PDS intensity evolution (Figure 3b–d). At a time delay of approximately 10 ps, depending on the particular **q′** point, the e-ph and ph-ph energy flows in the system becomes comparable (Figure 4). As a result, the population of strongly e-ph coupled modes is prevented from increasing further, as shown in the mode-resolved PDS (see Figure 4i–l). Losses through phonon scattering processes begin to dominate in these modes and reduce their population. Such dynamics may result in a local minimum in the PDS evolution, depending on the mode’s contribution to the total PDS intensity (e.g., Figure 3a, Figure S5a, Figure S6 vs Figure 4i). The PDS as a function of **q′** is highly sensitive to the population of certain phonon modes. This sensitivity is a direct result of the one-phonon structure factor (eq 5 and Figure 4a–h). Therefore, the dynamics of several phonon modes is not reflected directly in the PDS intensity, and the energy flow can be traceless in the experimental geometry. Regarding the studied **q′** points, the TA_2_ mode’s dynamics is dominant inside the BZ (Figure 4m–p and Figure S3). However, close to the BZ border, the contributions from the optical modes become essential. This is the only part of the investigated **q′** space where they drive the PDS evolution (Figure 3d, Figure S5d, and Figure 4l).

The different coupling parameters governing the e-ph energy flow and ph-ph scattering to a particular mode can, at specific **q′** points, result in a reversal of the rate of change of the mode population, even long after laser excitation. Such a mechanism can explain the occurrence of a local minimum in the evolution of PDS. Our simulations show that when considering **q′** = (0,–1/8,1/8), the TA_2_ mode (dominating PDS) is depopulated via phonon scattering processes that will overtake the contribution from the e-ph flow at approximately 7 ps (Figure 4e,i). Consequently, the TA_2_ contribution to the PDS intensity will decrease (Figure 4i). However, at long time scales, a combination of persistent residual e-ph energy flow (Figure 4e) and diminishing, or after 29 ps even reversing, contribution from ph-ph scattering (Figure 4a) will result in an increase in simulated PDS intensity after passing a local minimum at around 21 ps. This is in agreement with the experimental observation (Figure 3a, Figure S5a, and Figure S6).

We note that the experimental observation of a minimum in the PDS time evolution is located at a **q′** position close to the Γ point (Figure 3a, Figure S5a, and Figure S6). From our simulations (Figure 4), we can deduce that the TA_2_ mode becomes more highly populated near the Bragg spot (Γ point), compared to other parts of the BZ, and that this mode is more strongly coupled to the electronic subsystem than other modes important for the observed PDS intensity (Figure 3, Figures S5 and S6). This contributes to conditions favorable for the observation of a minimum (Figure 3a, Figure S5a, and Figure S6). At **q′** positions further away from the Bragg spot, the disproportion in the induced phonon mode populations is less significant, which results in the population of a single mode becoming less dominant to the PDS intensity (Figure 3b–d, Figure S5b–d). A similar PDS evolution was recently reported by Cotret et al.,^18^ with results including slower PDS rise times at increasing distance from the Bragg spot. In a similar vein to our work, this study also reported a significant influence of e-ph coupling on the system. The authors observed a two-component behavior, which was empirically explained by fitting the experimental results to a model that included the formation of two polarons of different sizes.^18^ In contrast, our framework considers the time-dependent phonon population driving the PDS intensity, and our results indicate that the dynamics are more complex and strongly dependent on both the **q′** location and mode-dependent e-ph and ph-ph coupling. Our analysis allows us to identify the most significant phonon modes and to track their dynamics. Within 7 ps of time delay, we find the onset of PDS intensity mostly driven by e-ph coupling resulting from a prompt population of the phonon modes. Note that at longer pump–probe delay times the contribution from ph-ph scattering starts to dominate over e-ph scattering. This results in a saturation of the PDS intensity (Figure 3b–d and Figure S5b–d). Depending on the coupling at specific **q′** positions, a complex interplay can give rise to local PDS minima that were experimentally observed at the time delays investigated in the present study.

### Umklapp Scattering and Thermal Conductivity

The ph-ph interactions in our simulations are related to three-phonon scattering processes, which can be divided into momentum conserving (normal) and nonconserving (Umklapp) processes.^40^ Unlike normal scattering, Umklapp scattering processes contribute to the system’s thermal resistance as they do not conserve the phonon momentum.^41−43^ It directs the phonons to be scattered in different directions, resulting in a reduced heat transfer in a material system.

**Figure 5:**
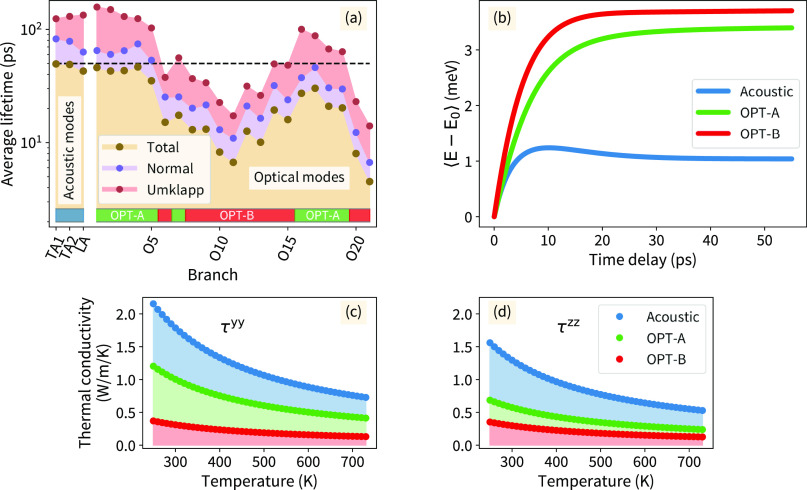
(a) Average mode-dependent phonon lifetime in the [100] zone. Normal and Umklapp scattering parts are resolved. Acoustic modes are labeled by their character (transversal acoustic-TA, longitudinal acoustic-LA), while the optical modes (OPT) are numbered in ascending frequency at Γ_(000)_ (O1–O21). (b) Averaged mode-resolved phonon energy gain over the [100] zone as a function of time. OPT-A includes branches (O1–O5, O7, and O16–19), and OPT-B includes the remaining optical modes. Mode-resolved thermal conductivity in the [100] zone: (c) τ^*yy*^ (d) τ^*zz*^ tensor components.

To estimate the amount of energy being subjected to the nonconservative ph-ph scattering processes, we combined the simulated nonequilibrium phonon populations with the calculated finite phonon lifetimes originating from the introduced three-phonon interaction (see Figure 5b). We evaluated mode-resolved phonon lifetimes at selected **q′** points to determine the influence of a phonon branch on the thermal properties. Our focus was on **q′** points lying in the [100] zone axis, same as in the experimental analysis. Furthermore, we considered only averaged values over the zone, which facilitates mode-dependent comparison (Figure 5a and Figure S10).

The results reveal that mainly the optical phonon branches around the phonon band gap (Figure 5a: modes O6–O15) and the highest-frequency branches (O20–O21) contribute to the nonconservative scattering processes. The related average Umklapp scattering lifetimes for these branches are below 20 ps, indicating a high scattering probability. These lifetimes are shorter than the lifetime of conserving processes belonging to some other phonon modes. Overall, these phonon modes bear a high probability of ph-ph scattering processes, as the averaged total lifetimes are on the order of 10 ps. Other optical and acoustic modes possess much longer normal as well as Umklapp scattering lifetimes, where the average value can exceed 100 ps (Figure 5a).

Unlike the situation in a simple single-atom system (e.g., Si^43^) where optical modes hardly contribute to the thermal transport, the calculated phonon band structure of the studied SnSe system indicates a more complex behavior (Figures S2A and S8). The optical phonon branches in SnSe cannot be considered as flat, and their non-negligible group velocity allows for heat transfer. However, they are subject to highly probable nonconservative scattering processes (Figure 5a). We divided the phonon modes into groups according to the magnitude of the Umklapp scattering lifetimes: the acoustic modes, optical modes with Umklapp lifetimes greater than 50 ps (OPT-A set), and optical modes with Umklapp lifetimes shorter than 50 ps (OPT-B set; Figure 5a). The artificial threshold of 50 ps was chosen based on the simulated time scale and the distribution of the Umklapp scattering lifetimes. It serves only as a guide for indicating bands with more probable nonconservative scattering. To verify the above statements, we calculated the related lattice thermal conductivity (Figure S11). The obtained tensor components along the lattice vector corresponds to other theoretical and experimental results.^9,14,44^ For a more detailed analysis, we focused on the **q′** points belonging to the [100] zone and extracted mode-dependent contributions (Figure 5c,d). The results demonstrate a high contribution from the optical phonon modes to the thermal conductivity (κ_OPT_^*yy*^∼0.73 κ_ACO_^*yy*^ and κ_OPT_^*zz*^∼0.67 κ_ACO_^*zz*^, where κ_OPT_, κ_ACO_ denotes optical and acoustic phonon mode contributions, respectively). Further decomposition of optical modes to the sets of OPT-A and OPT-B clearly demonstrates a low thermal conductivity contribution from the set of OPT-B modes. Considering the three phonon mode groups (acoustic, OPT-A, OPT-B), we evaluated related time-dependent phonon mode occupations (Figure S12) and the energy stored within phonon excitations (Figure 5b). This analysis revealed that the modes with short (OPT-B) and long (OPT-A and acoustic) Umklapp scattering lifetimes carry nearly the same amount of energy. This implies that during thermalization, the studied SnSe system tends to possess a significant population of excited optical phonon modes, which suffer remarkably from dissipative scattering processes (Figure 5a), leading to an increase in thermal resistivity^42,44^ (see Figure 5c,d). Nearly half of the energy in the excited phonon modes enters these modes in our simulation, which can explain the reported low thermal conductivity.^9^

## Conclusions

Achieving a comprehensive understanding of the energy dynamics of thermoelectric materials is essential for the design and development of highly efficient thermoelectric materials. In this pioneering study, we have presented fundamental insights into the low thermal conductivity of SnSe through a synergistic combination of experimental TDS and PDS analysis via ultrafast electron diffraction and theoretical description utilizing a nonequilibrium kinetic approach. Our findings offer a deeper understanding of the intricate interplay between the electronic and phononic subsystems, facilitating the rational design of advanced materials with tailored thermal properties.

We benchmarked our approach by analyzing experimentally measured TDS patterns. Our theoretical simulations enable us to explain the observed streak pattern formation in terms of crystal symmetry and attribute the contribution from relevant phonon modes.

To obtain a detailed understanding of the nonequilibrium phonon-mode dynamics, we conducted PDS experiments and subsequent momentum-dependent nonequilibrium kinetic theory analysis. We elucidate the flow of energy between the electron and phonon subsystems by comparing the experimental PDS to the simulated temporal dynamics at selected **q′** points. The PDS indicates a complex behavior including different onset of the PDS intensity, minima formation, or long-time scale behavior depending on the **q′**-position. From our nonequilibrium kinetic simulations, we identify e-ph and ph-ph energy flow processes with the calculated PDS features matching those observed in the experiment. Further, we deduce transient phonon occupations and associate these to nonequilibrium occupation of optical modes. Interestingly, nearly half of the energy in the excited phonon systems enters optical modes with a short Umklapp scattering lifetime, leading to a large thermal resistivity. Thus, by using a photoexcited electron system to mimic a doped thermoelectric material, we capture a detailed picture of the nonequilibrium phonon dynamics that explains the low thermal conductivity. Our study describes the nonequilibrium dynamics of phonon modes and their contribution to thermal transport, offering a perspective on energy transport in condensed matter. These results have far-reaching implications for the design of advanced materials with tailored thermal properties and are applicable to the dynamic description of quantum materials.

## Methods

### Experimental Details

The single-crystal SnSe sample was purchased from 2D Semiconductors, USA. To prepare the samples for the analysis, they were sliced parallel to the *b*-*c* plane of the crystal^39^ using a diamond knife mounted in a Leica Ultracut ultramicrotome. We observed that the sample is fragile, and it was difficult to prepare samples with thicknesses below 50 nm.

The thin-film samples were then placed on a single-layer graphene transmission electron microscope (TEM) grid (Ted Pella, USA). The ultrafast electron diffraction (UED) experiments were conducted at the Ultrafast Electron Microscopy (UEM) laboratory at the KTH Royal Institute of Technology, Sweden. A schematic illustration of the setup is presented in Figure S4a, and further details of the facility can be found in the paper by Ji et al.^38^ In brief, the UEM instrument operates in the pump (photons)-probe (photoelectrons) mode, as shown in Figure S4a. It consists of a modified JEOL JEM 2100 transmission electron microscope (TEM) with a thermionic electron gun operating at 200 keV. Ultrafast photoelectron pulses (probe) are generated via a photoemission process using excitation with femtosecond UV (∼4.8 eV) pulses on a guard ring LaB_6_ cathode. The full width at half-maximum (fwhm) of the probe pulses employed in the experiments had a temporal duration of around 1.2 ps, and this was determined using a technique called photoinduced near-field electron microscopy (PINEM),^45^ whereas a synchronized pump pulse of energy ∼2.4 eV and fwhm of ∼270 fs from the same laser source (Tangerine, Amplitude Systemes) were focused to a spot size of ∼120 μm on the sample to initiate the photoinduced processes. The relative time delay between the pump and photoelectrons probe pulses is controlled by the Newport Motion controller (model ESP301) delay stage. The time-resolved electron diffraction measurements were done at room temperature. The sample was photoexcited at 20 to 35 kHz repetition rate to allow for complete relaxation of the sample between excitation pulses. In the UED experiments and analysis, an ultrafast laser pulse (pump) is employed to drive the system into an excited electronic state. After a controlled time delay, the sample is probed by a photoelectron pulse, which diffracts from the lattice. The electron diffraction patterns, measured at different pump–probe time delays, provide a direct momentum-resolved probe of the nonequilibrium lattice dynamics of the system (Figure S4 and Figure 2). The SnSe sample was photoexcited with ∼270 fs laser pulses with a photon energy ∼2.4 eV (515 nm), resulting in an almost instantaneous excitation of electrons from the valence band to empty states in the conduction band (as the indirect bandgap of SnSe is 0.90 eV and the direct 1.30 eV).^46^

The temperature-dependent electron diffraction measurements were carried out using a Gatan double-tilt heating holder (model 652) from room temperature to 673 K. All experiments were conducted in the transmission geometry with the incident electron beam along the [100] zone axis. All of the diffraction patterns were collected using a highly sensitive Medipix3 detector from Amsterdam Scientific Instruments (ASI), Netherlands. The thickness of the sample was determined using electron energy loss spectroscopy (EELS). The atomic arrangements of SnSe were viewed using visualization for electronic and structural analysis (VESTA) software,^39^ whereas the Bragg spots in the electron diffraction patterns were identified using the SingleCrystal 3 from the CrystalMaker software Ltd., UK.

Notably, in order to improve the precision of the experimental PDS results, especially close to Bragg spots (see Figure 3a, Figure S5a, Figure S6a,b), we performed a drift correction of the UED patterns. The positions of the Bragg peaks have been corrected to 1/10 of a pixel precision through fitting of the peaks by a Gaussian function. The drift can be attributed to instabilities of the electromagnetic lens system of the TEM over the extended acquisition period of our PDS measurements.

### Computational Details

Ground-state geometries and properties were calculated using plane-wave-based density functional theory (DFT) with the VASP software.^47^ The generalized gradient approximation of Perdew, Burke, and Ernzerhof (PBE)^48^ was used for the exchange-correlation (xc) functional, and the core electrons were represented via scalar relativistic Projector Augmented Wave (PAW) pseudopotentials.^49^ van der Waals (vdW) corrections were introduced through the zero-damping DFT-D3 method of Grimme [DFT-D3].^50,51^ The plane-wave basis set had an energy cutoff of 500 eV, and the momentum space was sampled by a Γ-centered 6 × 11 × 11 k-point mesh. The atomic coordinates and cell size were optimized until the interatomic forces were smaller than 1 × 10^–6^ eV/atom. The first-principles calculation of the phonon modes was performed using the finite displacements method using VASP and phonopy.^52,53^ A 2 × 4 × 4 supercell including 256 atoms was used.

The coupled differential equations describing the nonequilibrium dynamics of the phonon systems, Equations 1 and (2), were solved numerically on an 8 × 8 × 8 **q**-grid in momentum space including all phonon branches. The ph-ph scattering matrix elements Γ_*μν*_(**q**, **k**) were calculated with VASP and phono3py^53,54^ using the same parameters used for the phonon simulations. The e-ph coupling parameters *γ*_*ν*_(**q**) were obtained using the EPW module^55^ included in the Quantum ESPRESSO (QE),^56^ a plane-wave-based DFT calculation suit. Based on the Maximally Localized Wannier Functions (MLWF),^57^ EPW allows significantly reduced computational demands of el-ph calculations. Similar to the previous VASP calculations, scalar-relativistic PAW pseudopotentials, GGA xc-potentials of PBE, and DFT-D2-based vdW corrections^58,59^ were involved in the ground-state electron structure calculations.^55,56^ They were performed on a uniform 8 × 8 × 8 k-mesh with a plane-wave basis energy cutoff of ∼800 eV (60 Ry). The atomic coordinates were relaxed reaching interatomic forces lesser than ∼2 × 10^–5^ eV/Å (10^–6^ a.u). In order to employ the EPW module, dynamical matrices were obtained through the density functional perturbation theory approach as implemented in the QE, where a 4 × 4 × 4 q-mesh was employed. Working with the MLWF, the e-ph coupling and e-ph lifetimes were calculated along a path connecting high-symmetry k-points using the electron structure interpolated to a denser 24 × 24 × 24 k-mesh. The e-ph calculations were performed for various Fermi level (*E*_F_) positions lying in the vicinity of the energy band gap to describe an impact of the *E*_F_ on the obtained results. The calculated e-ph coupling parameters *γ*_*ν*_(**q**) resulted in a too-rapid PDS response. Therefore, the parameters were rescaled to follow the experimentally observed dynamics (see the main text).

In addition, the lattice thermal conductivity was evaluated with phono3py via the linearized Boltzmann phonon equation.^53,54^ To analyze the Umklapp scattering,^40^ phonon lifetimes, and lattice thermal conductivity, an extra 20 × 40 × 40 k-mesh was considered in the phono3py calculations.

### Nonequilibrium Kinetic Theory

To describe the transient evolution of the diffuse scattering, we used an out-of-equilibrium kinetic theory developed to monitor the nonequilibrium energy flow between the electronic system and the wave-vector and branch-dependent phonon modes.^33−35^ This model is based on total energy conservation and on a semiclassical kinetic theory and provides the transient dynamics of the nonequilibrium phonon population following ultrafast laser excitation. Importantly, it includes an explicit dependence of anharmonic effects, describing ph-ph interactions and of the e-ph scattering on the wave-vector and phonon branches. As a result, a set of rate equations are defined providing the time evolution of the nonequilibrium energy flow between the electronic () system and the different phonon modes)
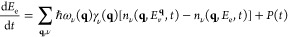
1
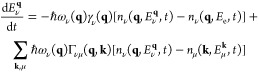
2for all the wave-vectors **q** and branches ν. *n*_*ν*_(**q**, *E*_*ν*_**^q^**, *t*) is the out-of-equilibrium phonon population of phonon mode **q** with branch *ν*, with *E_ν_***^q^** being the time-dependent amount of energy stored in this particular mode. *ω*_*ν*_(**q**) is the frequency of the phonon mode with reduced wave-vector **q** and branch ν. *γ*_*ν*_(**q**) and Γ_*νμ*_(**q**, **k**) are the phonon line widths due to e-ph and ph-ph scattering, respectively, which depend explicitly on the phonon mode. *P*(*t*) is the pump-laser field that generates the nonequilibrium electronic distribution. Note that while the first term on the right-hand side of eq 2 defines the energy flow due to e-ph interaction, the second term accounts for the energy flow due to ph-ph scattering processes.

The numerical solutions of eqs 1 and (2) give us access to the time evolution of the nonequilibrium phonon populations, which explicitly determine the transient diffuse scattering, defined as^36^
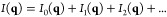
3where the first term on the right-hand side corresponds to the Bragg diffraction and the other terms correspond to first-order diffuse scattering, second-order diffuse scattering, etc. It is important to note that unlike the thermal case these terms explicitly depend on the time delay defined after laser excitation. In this work, we focus on the first-order term, which is the one that dominates the contribution to the diffuse scattering. We also compute the second-order term and find that it is at least 2 orders of magnitude smaller than the first order. Thus, we compute^36^
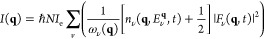
4with
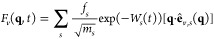
5where *I*_e_ is the intensity of scattering from a single electron, *N* is the number of primitive cells in the crystal (*N* = 1 in the simulations), *F*_*ν*_ is one-phonon structure factor, *f*_*s*_ is the atomic scattering factor of the *s*-th atom in a unit cell and *m*_*s*_ denotes its mass, *W*_*s*_ is the DW factor, and **ê**_*v*,*s*_ is the polarization vector. The polarization vectors out of the first BZ are modified by a phase factor^36^
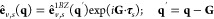
6where **q′** is the relative position of the scattering vector **q** with respect to the nearest Bragg spot **G**, and **τ**_*s*_ stands for an atomic basis vector within the unit cell.

We note that the considered first-order TDS approach produced an artificial step in the simulated TDS intensity at the BZ boundaries (Figure1b,e). It originates from a sudden change of vector **G** (eq 6), which is constant for all **q**-points across a certain BZ.

## Supporting Information Available

PNMA crystal structure of layered SnSe; phonon spectrum of SnSe, including phonon bands with and without the nonanalytical correction (NAC); comparison of TDS patterns obtained from calculations with and without the NAC; analysis of TDS line profiles, with and without the NAC; phonon branch-resolved simulated TDS; additional UED results; momentum-resolved nonequilibrium phonon dynamics; comparative analysis of experimental photoinduced diffuse scattering (PDS) with theoretical models with varying electron–phonon coupling parameter; examination of phonon band characteristics as a function of **q**-position, considering cases with and without the NAC; investigation of the relationship between electron–phonon (e-ph) and phonon–phonon (ph-ph) energy flows and their contribution to the total PDS intensity; phonon branch-dependent lifetimes; lattice thermal conductivity tensor; phonon mode-resolved mean population differences in the [100] zone as a function of time (PDF)
