# Supplementary material for: Nonequilibrium Phonon Dynamics and Its Impact on the Thermal Conductivity of the Benchmark Thermoelectric Material SnSe
Source: ACS Nano. 2023 Oct 20;17(21):21006–17. doi: 10.1021/acsnano.3c03827 (PMC10655201; doi:10.1021/acsnano.3c03827)
Supplement: Supplementary file 1 — nn3c03827_si_001.pdf [file nn3c03827_si_001.pdf]

Supporting Information (SI)

Nonequilibrium phonon dynamics and its impact on the thermal conductivity of the benchmark thermoelectric material SnSe

Amit Kumar Prasad[1†], Jakub Sebesta[2†], Raquel Esteban-Puyuelo[2], Pablo Maldonado[2], Shaozheng Ji[1], Biplab Sanyal[2], Oscar Grånäs[2], and Jonas Weissenrieder[1*]

[1]Materials and Nano Physics, School of Engineering Sciences
KTH Royal Institute of Technology, SE-100 44 Stockholm, Sweden

[2]Materials Theory, Department of Physics and Astronomy, Uppsala University
Box 516, 751 20 Uppsala, Sweden

(*Corresponding Author: J.W., E-mail: jonas@kth.se)

(† Authors contributed equally to this work)

Table of contents

Figure	Description	Page
**S1**	Pnma crystal structures of layered SnSe in different crystallographic directions.	2
**S2(A)**	Phonon spectrum of SnSe along its high-symmetry points. Phonon bands without and with the non-analytical correction (NAC).	3
**S2(B)**	TDS patterns comparison for calculation without and with the non-analytical correction (NAC).	4
**S2(C)**	TDS line profile comparison without and with the non-analytical correction (NAC).	4
**S3**	Phonon branch-resolved simulated thermal diffuse scattering (TDS).	5
**S4**	Ultrafast electron diffraction (UED) measurements of SnSe.	6
**S5-6**	Momentum resolved nonequilibrium phonon dynamics.	7-8
**S7**	Comparison of experimental PDS with theoretical models with varying e-ph coupling parameter (Ge-ph).	9
**S8**	Phonon band character as a function of q-position for without and with the non-analytical correction (NAC).	10
**S9**	Relation of the e-ph and ph-ph energy flows to the phonon modes contributing the total PDS intensity.	11
**S10**	Phonon branch dependent lifetime of imaginary part of the Umklapp ph-ph scattering process self-energy along the [100] zone axis.	12
**S11**	Lattice thermal conductivity tensor.	13
**S12**	Mode-resolved mean population difference in the [100] zone as a function of time.	14

***Pnma* crystal structures of layered SnSe in different crystallographic directions**

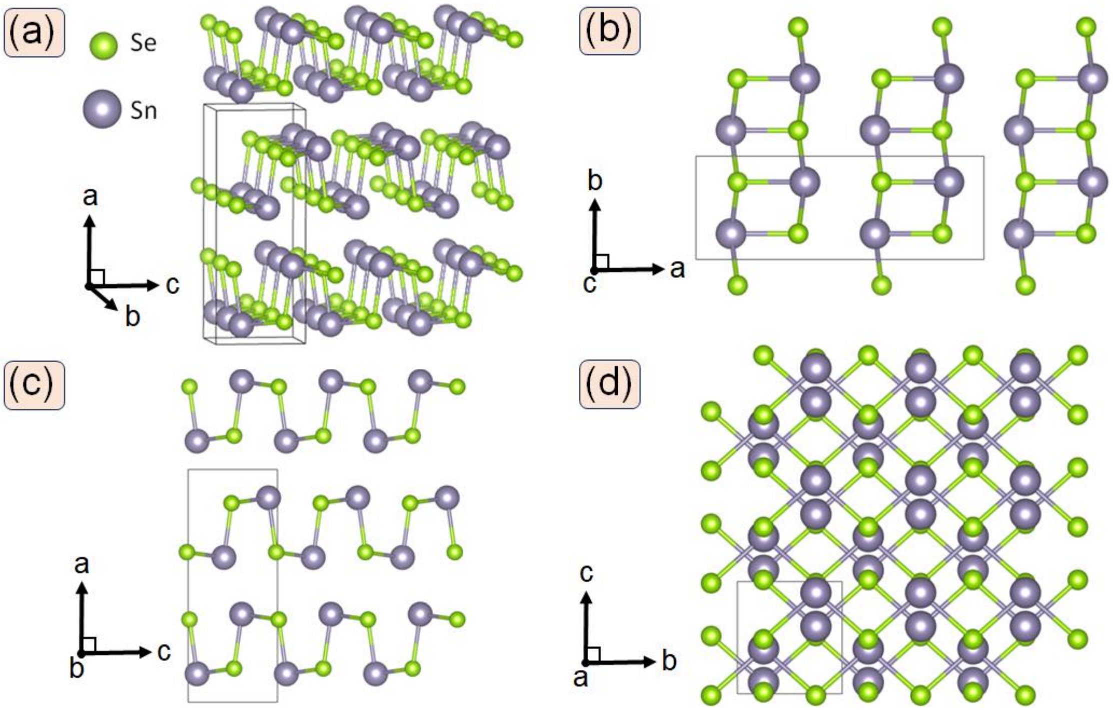

Figure S1. Crystal structure of SnSe in the *Pnma* phase projected in (a) three dimensions, (b) along the **c** axis, (b) along the **b** axis, and (d) along **a** axis. Color codes: gray (Sn) and green-yellow (Se). The structures were created using the Visualization for Electronic and Structural Analysis (VESTA) software.[1]

Phonon spectrum of low temperature Pnma phase of SnSe

We calculated the phonon band structure and phonon density of states of bulk SnSe, and the results are plotted in Figure S2A. The lowest frequency branches are mostly contributed by Sn atoms and are separated by a gap from the high-frequency ones, dominated by Se atoms.

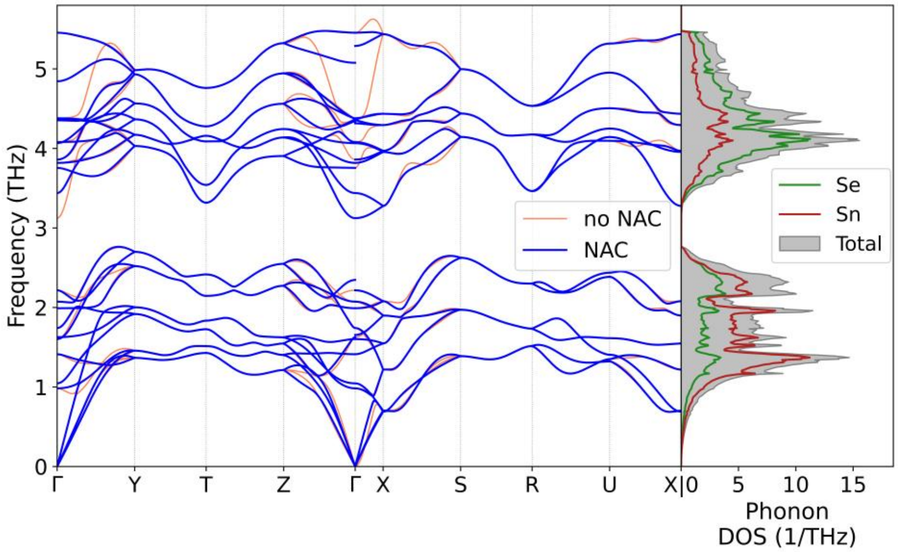

Figure S2 A. Left - Phonon spectrum of SnSe along its high-symmetry points. Phonon bands without and with the non-analytical correction (NAC) are depicted. Right - total phonon density of states shown in shaded grey, and projection to the Sn and Se atoms in red and green lines, respectively. NAC is considered.

Having calculated the TDS, we compared the result given by phonon spectra with and without non-analytical correction (NAC) describing a correction arising from macroscopic field caused by atomic displacement induced polarization of non-metallic materials.[2] The NAC brings modification to the phonon spectra (Figure S2A), particularly the presence of polar phonons,[3,4] and related phonon band shifting. Despite the change in phonon spectra, the TDS shown in Figure 1(b) of the main text is almost independent of the application of NAC as the TDS patterns with and without NAC are hardly distinguishable (Figure S2B). Similarly, the implication of NAC should be considered for the lineprofile in Figure 1(f) (main text) that analyze the TDS between the $\Gamma_{(020)}$– $\Gamma_{(011)}$ points. Figure S2 C show that the total TDS curves with or without NAC are identical and differences in the mode contributions are marginal. This indicates that the modifications of the phonon spectrum, occurring especially for higher optical phonons, are irrelevant for the observed TDS patterns. The mode resolved contributions prove that the dominant contributions are provided by the lowest lying (Figure S2 C) acoustic contribution (Figure S3), where only near the Brillouin zone edge the optical mode contribution can play role as line profiles indicate. The analysis in the main article considers data including the NAC.

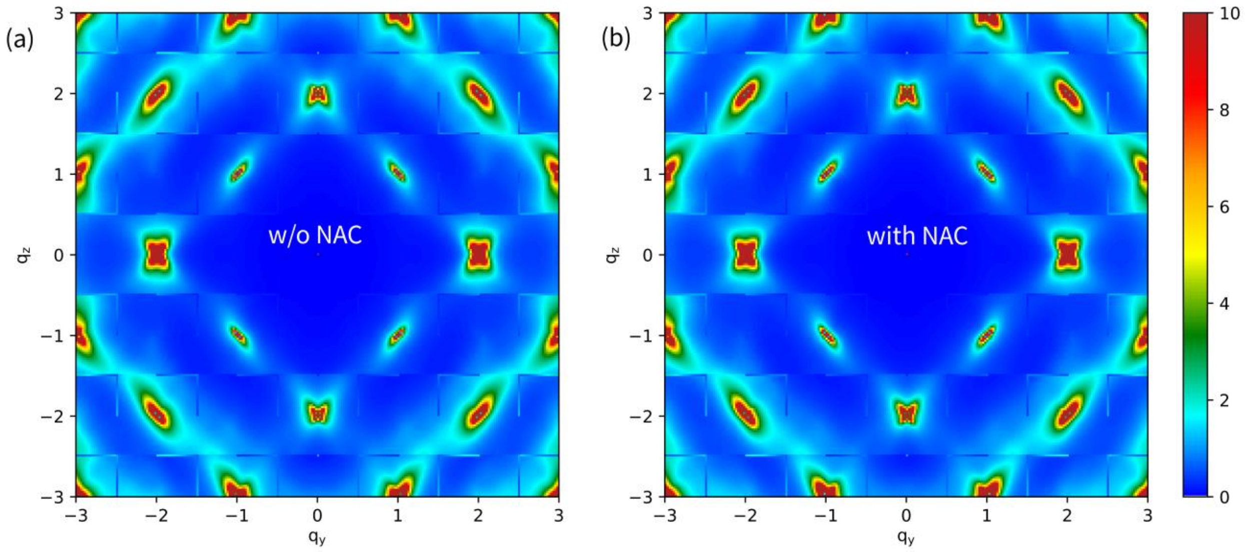

Figure S2 B. TDS patterns comparison for calculation (a) without and (b) with the non-analytical correction (NAC).

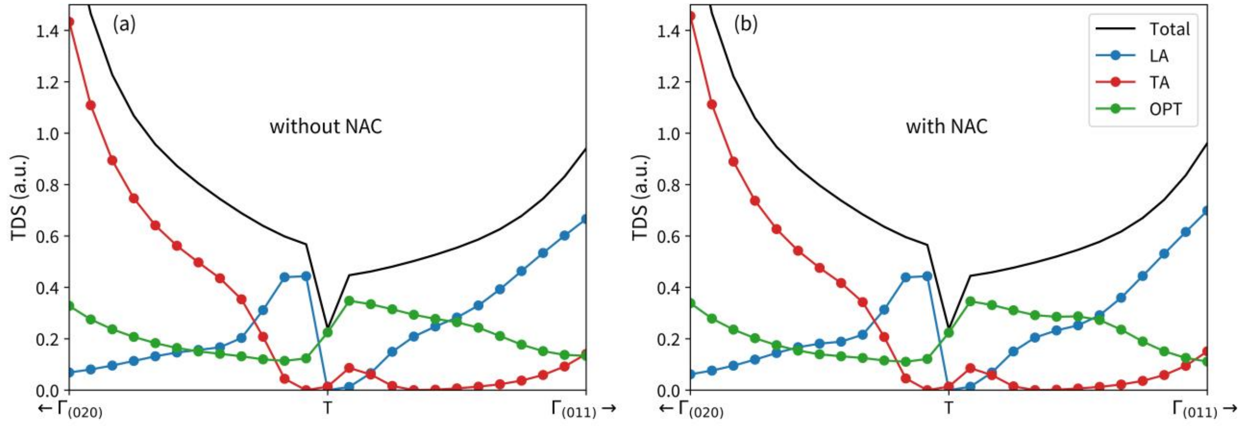

Figure S2 C. TDS line profile comparison (a) without and (b) with the non-analytical correction (NAC). The regions extracted for line of profiles are indicated by white rectangles in the Figure S2B).

Phonon branch-resolved simulated thermal diffuse scattering (TDS)

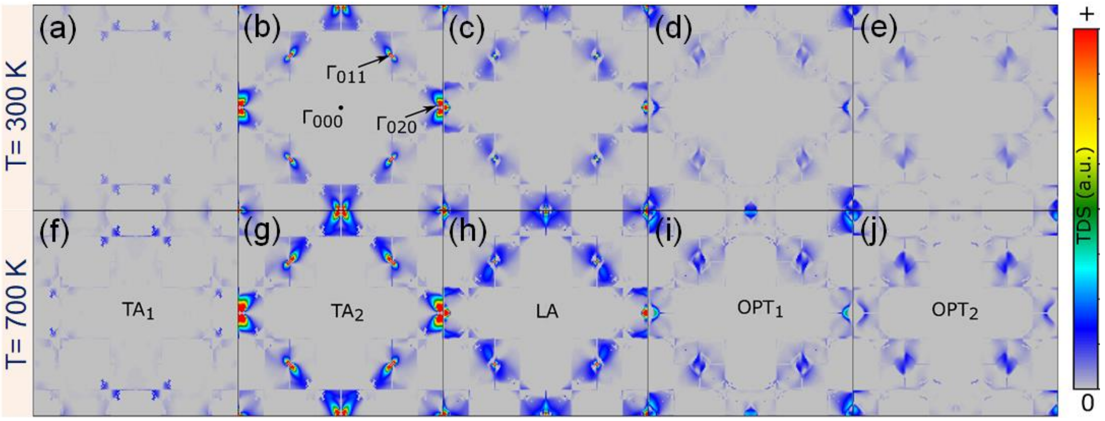

Figure S3. Calculated phonon mode dependent thermal diffuse scattering (TDS) along the [100] zone axis of SnSe. (a-e) TDS at 300 K, and (f-j) TDS at 700 K. The contribution of individual phonon modes to the TDS is presented in figure: (a, f) Transversal acoustic TA_1, (b, g): TA_2, (c, h) Longitudinal acoustic (LA), (d, i) and (e, j) Optical modes (OPT).

Ultrafast electron diffraction (UED) measurements of SnSe

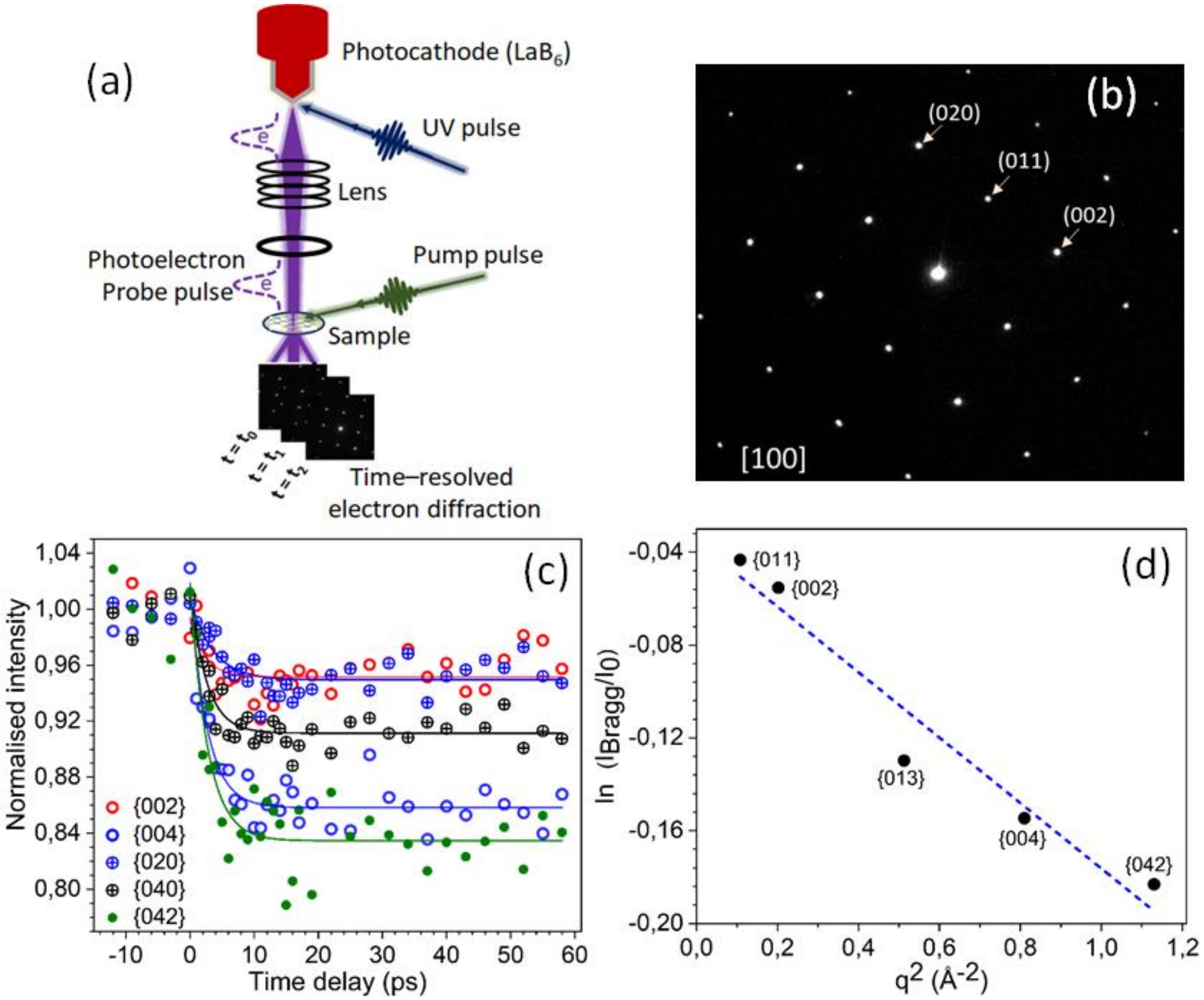

Figure S4. (a) Schematic illustration of ultrafast electron diffraction (UED) in an Ultrafast electron microscope (UEM). (b) Representative electron diffraction pattern from the *Pnma* phase of SnSe in the [100] zone axis. (c) Time evolution of Bragg diffraction intensity for five family of Bragg diffraction spots for a 300 fs incident pump at ~ 2.4 eV with a fluence of 2.4 mJ/cm^2. (d) Natural logarithm plot of measured normalized Bragg peak intensities averaged between the data at time delays 30 to 54 ps for different Bragg Spots shown as black solid circles), as a function of the square of reciprocal vector, **q**. The linear dependence suggests the Debye−Waller behavior, as shown in figure (d).

Momentum resolved nonequilibrium phonon dynamics

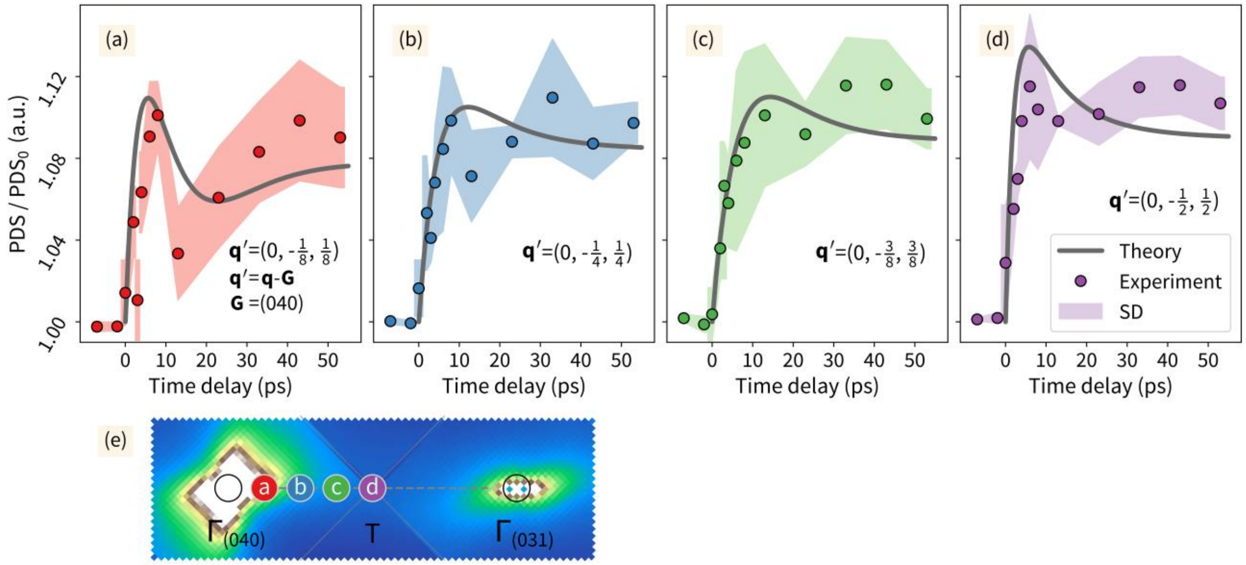

Figure S5. Momentum resolved temporal dependence of experimental photoinduced diffuse scattering (PDS) in comparison with nonequilibrium kinetic theory simulations. Relative change in PDS (normalized to PDS_0 collected at time delays < 0 ps). Results were analyzed at four different momentum-space positions: (a) **q´** = (0, -1/8, 1/8), (b) **q´** = (0, -1/4, 1/4), (c) **q´** = (0, -3/8, 3/8) and (d) **q´** = (0, -1/2, 1/2) along the Γ(040) – T – Γ(031) path. Here, experimental PDS (data points), standard deviations (SD, colored regions) of the experimental data points and simulated PDS (solid lines) are represented. Model T_4: $G_{e\text{-}ph}$ (see Figure S7) was used for this simulated PDS, (e) Positions of the selected **q´**-points projected on the simulated PDS pattern at time delays < 0.

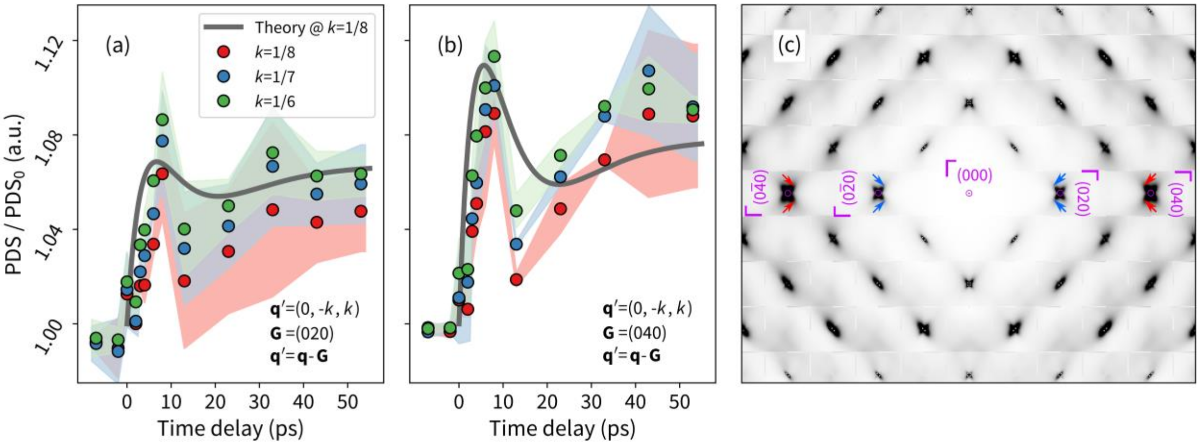

Figure S6. Illustrates the momentum-resolved temporal dependence of experimental photoinduced diffuse scattering (PDS) in comparison with nonequilibrium kinetic theory simulations. The relative change in PDS is normalized to PDS_0 at time delays < 0 ps. The analysis was conducted at positions close to Bragg spots at three distinct momentum-space positions: **q´** = (0, -1/8, 1/8), (0, -1/7, 1/7), and (0, -1/6, 1/6) in (a) near (020) along $\Gamma_{(020)}$ – T – $\Gamma_{(011)}$, and (b) near (040) along $\Gamma_{(040)}$ – T – $\Gamma_{(031)}$, (c) Symmetric k-space regions used for averaging the experimental PDS data indicated by blue arrows near (020), $(0\bar{2}0)$ and red arrows close to the (040), $(0\bar{4}0)$ Bragg spots. At each of these selected **q´** points, the experimental data were integrated, over an area of 0.67 x 10^{-4} $Å^{-2}$ and then averaged over symmetrical **q´** points. Model T_4: $G_{e\text{-}ph}$ (see Figure SI 7) was used for this simulated PDS, Here, experimental PDS (data points), standard deviations (SD, colored regions) of the experimental data points and simulated PDS (solid lines) are represented.

Figure 3(a) and Figure S5(a) focus on comparing the experimental and simulated PDS data at **q´** = (0, -1/8, 1/8). We have expanded our analysis to include PDS data for **q´** = (0, -1/7, 1/7) and (0, -1/6, 1/6) in Figure S6(a) and (b). These intermediate points within the experimental pattern exhibit a similar trend in the 10 to 30 ps range to **q´** = (0, -1/8, 1/8).

Comparison of experimental PDS with theoretical models with varying e-ph coupling parameter ($G_{e\text{-}ph}$)

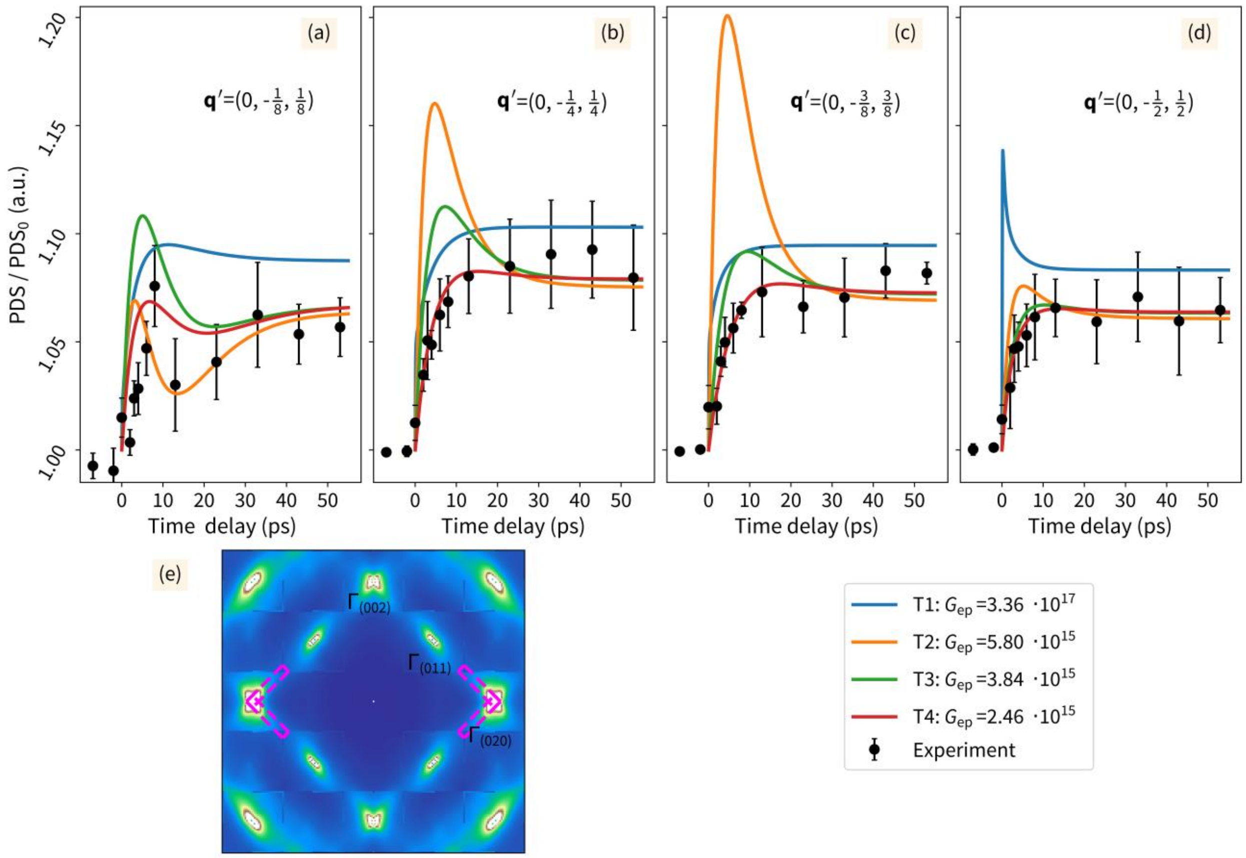

Figure S7. Comparison of the simulated photoinduced diffuse scattering (PDS) data with the experimental results. The relative change of the PDS with respect to the PDS at pump probe time delay < 0 ps is depicted. The data were analyzed at the four selected **q´** -points along the $\Gamma_{(020)}$ –T – $\Gamma_{(011}$ direction: (a) **q´** = (0, -1/8, 1/8) *i.e.* **q´** at 12.5 % of the $\Gamma_{(020)}$– $\Gamma_{(011)}$ distance, (b) **q´** = (0, -1/4, 1/4) *i.e.* **q´** at 25 % of the $\Gamma_{(020)}$– $\Gamma_{(011)}$ distance, (c) **q´** = (0, -3/8, 3/8) *i.e.* **q´** at 37.5 % of the $\Gamma_{(020)}$– $\Gamma_{(011)}$ distance, and (d) **q´** = (0, -1/2, 1/2) *i.e.* **q´** at 50 % of the $\Gamma_{(020)}$– $\Gamma_{(011)}$ distance. Simulated PDS for different sets of e-ph coupling parameters (G_{ep}) in W/m^3 K (color solid lines). Related e-ph energy transfer coefficients G_{ep} are given in the box. Experimental data are shown as black data points with error bars denoting their standard deviations. (e) Simulated TDS pattern with depicted symmetric regions (magenta rectangles) used for experimental data averaging.

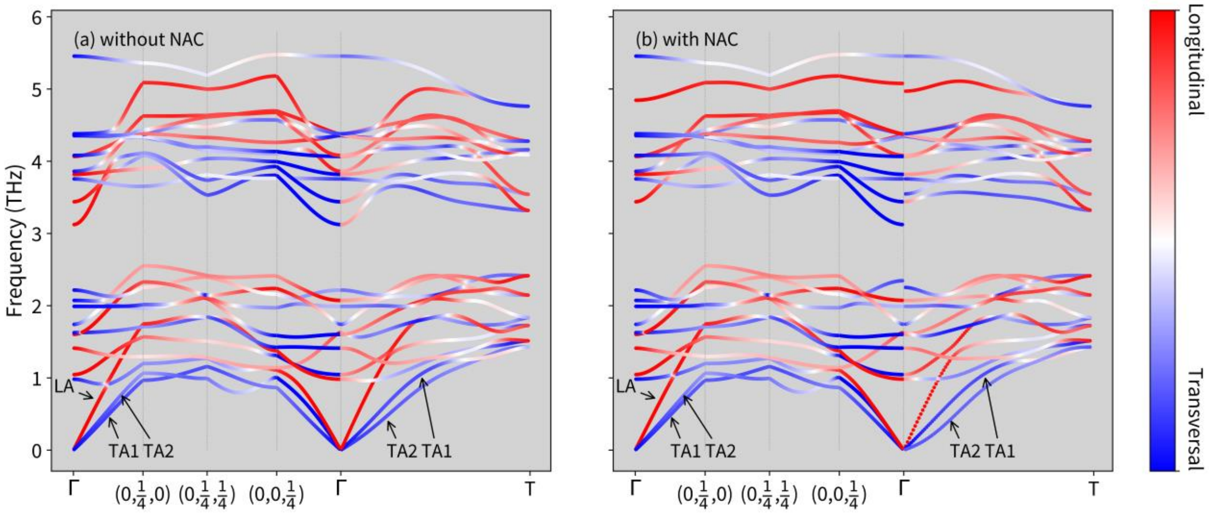

Figure S8. Phonon band character as a function of **q**-position along a path in the [100] zone for (a) without and (b) with the non-analytical correction (NAC). The color of the solid lines denotes the mean value of the phonon eigenvectors' projection to the direction of the **q**-vector, assuming eigenvectors related to all atoms in the unit cell.

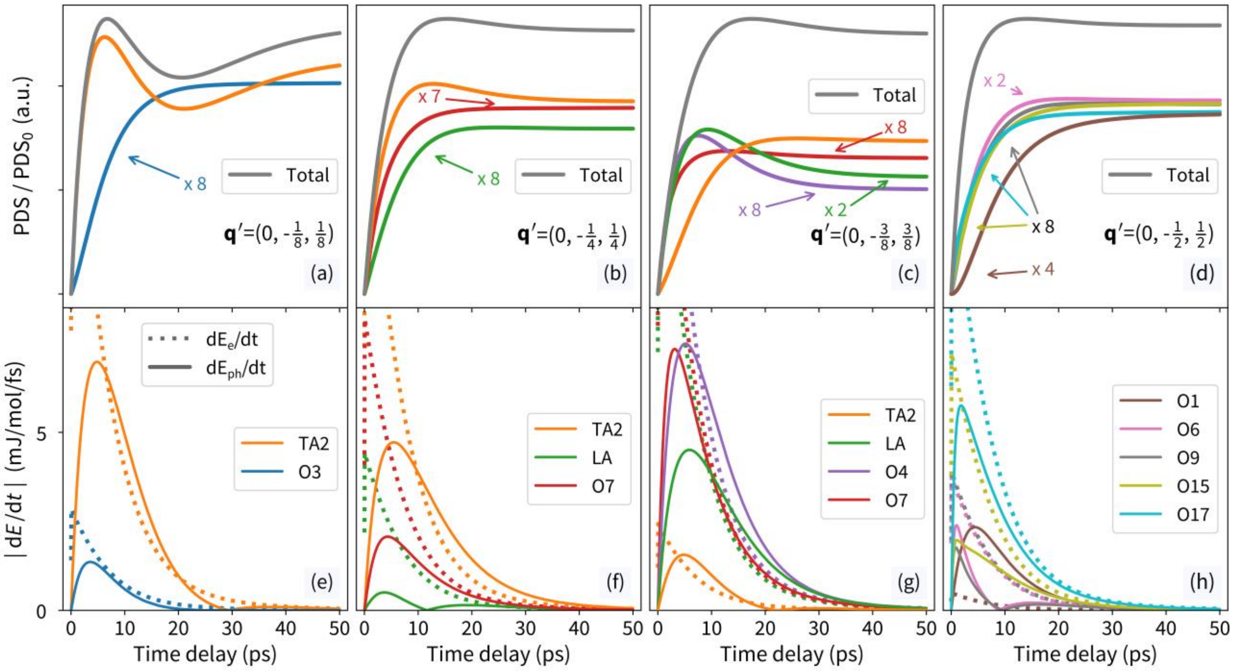

Figure S9. Relation of the e-ph and ph-ph energy flows to the phonon modes contributing the total PDS intensity. (a-b) The relative change of the PDS with respect to the PDS at pump probe time delay < 0 ps is depicted. Total PDS intensity and dominant phonon mode contributions are depicted. (e-h) Comparison of the absolute values of the e –ph (colored dashed lines) and ph-ph (colored solid lines) energy flows related to the selected modes in (a-b).

Figure S9 contains a direct comparison between the dE_{ph}/dt and dE_e/dt flows for the selected branches, offering insights into the dominant energy flow at specific times. Furthermore, the resulting mode contribution to the PDS are compared to the total simulated PDS.

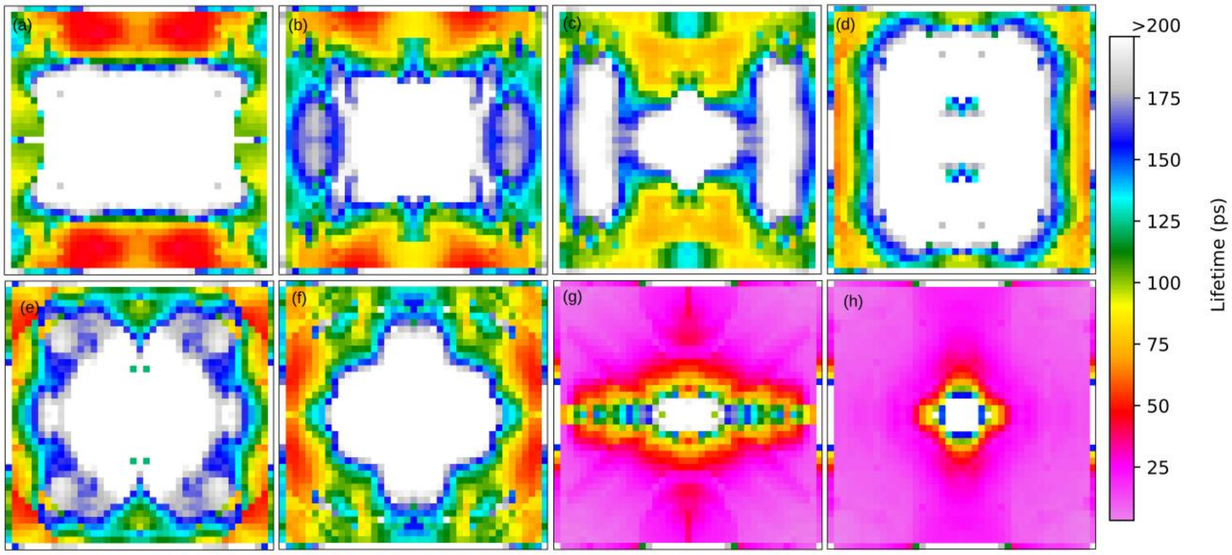

Figure S10. Phonon branch dependent lifetime of imaginary part of the Umklapp ph-ph scattering process self-energy along the [100] zone axis. The color bar denotes the phonon lifetime, (a) to (c) acoustic phonons, (d) to (h) Optical phonons.

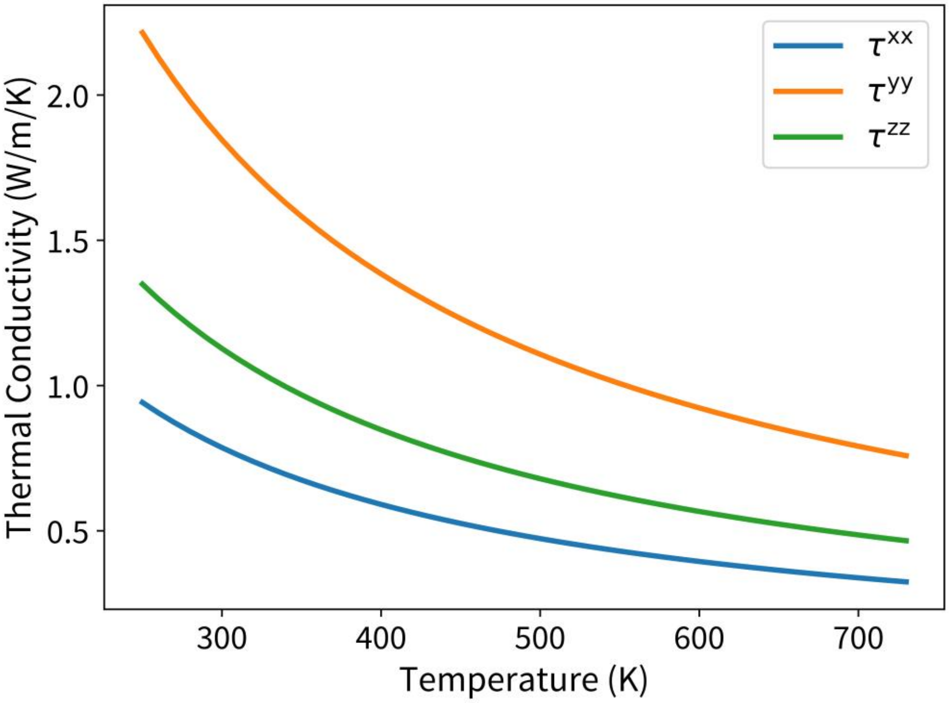

Figure S11. Lattice thermal conductivity tensor. The diagonal elements are depicted.

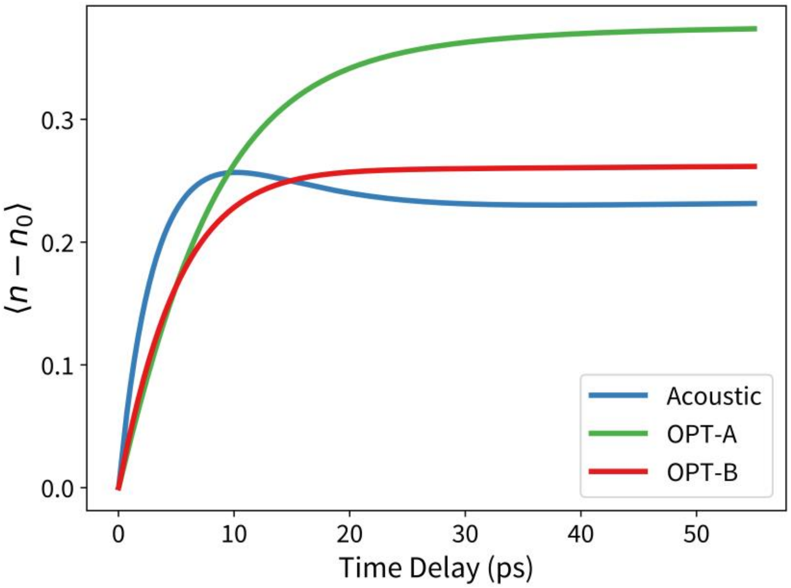

Figure S12. Mode-resolved mean population difference in the [100] zone as a function of time. Acoustic and optical modes are resolved. OPT-1 includes optical branches (O1-O5, O7, O16-19), and OPT-2 includes the remaining optical modes with short phonon lifetimes.

To analyze the contribution of particular phonon modes to the thermal transport, we divided the phonon modes into three sets according to their character and the importance of the Umklapp scattering (Figure 5(a)). We evaluated the mean change of the phonon population (Figure S12) induced by the laser pulse for each of the selected phonon sets as follows:

$$\langle n - n_0 \rangle = \frac{1}{N_q} \frac{1}{N_m} \sum_q \sum_m \left(n_{qm} - n_{qm}^0 \right) \ , \tag{1}$$

where N_q denotes total number of the q-points q, N_m is number of phonon modes m in the mode set S (acoustic, opt-A, opt-B), n_{qm} stands for the time dependent phonon population of the mode m at the q-point q and n_{qm}^0 is initial phonon population. Then, the energy accumulated in those phonon-mode sets (Figure 5(b)) is obtained from the phonon spectral dependence:

$$\langle E - E_0 \rangle = \frac{1}{N_q} \frac{1}{N_m} \sum_q \sum_m \hbar\omega_{qm} \left(n_{qm} - n_{qm}^0 \right) \ , \tag{2}$$

where $\hbar\omega_{qm}$ stands for the energy of phonon mode m at the **q**-point $\boldsymbol{q}$.

References

1. Momma, K.; Izumi, F. VESTA 3 for Three-Dimensional Visualization of Crystal, Volumetric and Morphology Data. *Journal of Applied Crystallography* **2011**, 44, 1272– 1276.
2. Pick, R.; Cohen, M.; Martin, R. Microscopic Theory of Force Constants in the Adiabatic Approximation. *Physical Review B* **1970**, 1, 910– 920.
3. Caruso, F.; Troppenz, M.; Rigamonti, S.; Draxl, C. Thermally enhanced Fröhlich coupling in SnSe. *Physical Review B* **2019**, 99 (8), 081104.
4. Ma, J.; Chen, Y.; Li, W. Intrinsic phonon-limited charge carrier mobilities in thermoelectric SnSe. *Physical Review B* **2018**, 97, 205207.
